# Pepper Mild Mottle Virus: An Infectious Pathogen in Pepper Production and a Potential Indicator of Domestic Water Quality

**DOI:** 10.3390/v15020282

**Published:** 2023-01-19

**Authors:** Kingsley Ochar, Ho-Cheol Ko, Hee-Jong Woo, Bum-Soo Hahn, Onsook Hur

**Affiliations:** 1 National Agrobiodiversity Center, National Institute of Agricultural Sciences, Rural Development Administration (RDA), Jeonju 54874, Republic of Korea; 2 Plant Genetic Resources Research Institute, Council for Scientific and Industrial Research, Bunso P.O. Box 7, Ghana

**Keywords:** pepper mild mottle virus, nucleotide sequence, PMMoV, resistance, *Tobamovirus*, *Virgaviridae*

## Abstract

Pepper (*Capsicum* spp.; Family: Solanaceae; 2n = 24) is an important crop cultivated worldwide for the consumption of its fresh and dried processed fruits. Pepper fruits are used as raw materials in a wide variety of industrial processes. As a multipurpose vegetable crop, there is a need to increase the yield. However, yield productivity of pepper is severely constrained by infectious plant pathogens, including viruses, bacteria, fungi, and oomycetes. The pepper mild mottle virus (PMMoV) is currently one of the most damaging pathogens associated with yield losses in pepper production worldwide. In addition to impacts on pepper productivity, PMMoV has been detected in domestic and aquatic water resources, as well as in the excreta of animals, including humans. Therefore, PMMoV has been suggested as a potential indicator of domestic water quality. These findings present additional concerns and trigger the need to control the infectious pathogen in crop production. This review provides an overview of the distribution, economic impacts, management, and genome sequence variation of some isolates of PMMoV. We also describe genetic resources available for crop breeding against PMMoV.

## 1. Introduction

Vegetable crops are rich sources of basic food nutrients, including vitamins, minerals, and dietary fiber, as well as several antioxidant compounds required for human health [1]. Pepper (*Capsicum* spp.; family: Solanaceae) is one of the most important vegetable crops globally, and is widely cultivated for the consumption of its fruits, either fresh, dehydrated, or processed in spicy condiments [2]. As a versatile crop, pepper is widely cultivated in diverse climatic conditions in both fields and protected environments. At the global and regional scales, fresh pepper fruits and processed products are highly traded, making cultivation of the crop an important source of employment for many people, especially smallholder farmers around the world [3]. The crop is in demand for industrial uses, especially for the extraction of useful volatile molecules or compounds, including capsaicin, carotenoids, and tocopherol compounds, and for use as an ingredient in agrofoods, cosmetics, food preservatives, additives, antimicrobial preparations, and pharmaceuticals [2]. Therefore, it is important to increase the pepper fruit yield. However, the success of pepper cultivation in relation to productivity of the fruits is influenced by environmental conditions, which can place stress on the crop, leading to fruit yield loss and quality reduction [4]. Abiotic and biotic conditions are major environmental threats that limit the yield of vegetable crop species, and efforts to control the associated impacts usually require an investment of resources leading to increased production costs. Fruit yield loss in pepper continues to be recorded in many growing areas, which is largely attributed to a wide array of phytopathogens [5]. Pepper plants are susceptible to different plant pathogens, including viruses, bacteria, fungi, nematodes, oomycetes, and viroids. Plant viruses are economically important pathogens that are responsible for severe yield reduction and reduced marketable fruit quality, especially deformed fruits in cultivated pepper species such as *C. annuum*, *C. frutescens*, *C. chinense*, and *C. chacoense*. Among the various groups of viruses, members of the genus *Tobamovirus* in the family *Virgaviridae* are among the most deleterious pathogens, and account for injurious yield losses and reduced fruit quality in pepper production worldwide [5,6]. Pepper mild mottle virus (PMMoV) is a frequently detected plant virus in domestic and aquatic water resources as well as in human and other animal excreta, and it has therefore been suggested as a potential indicator for assessing global water quality [7,8]. The cultivation of pepper genotypes with inherent or conferred resistance against pathotypes of PMMoV is one of the best approaches for enhancing crop yields. Previous studies of pepper identified the L locus as containing allelic genes (L1–L4) for resistance against PMMoV in pepper plants [9]. However, the resistance of pepper plants to the virus differs between viral strains [10], and as the diversity of viral strains coupled with the emergence of more virulent strains or exposure to higher concentrations of the virus can overcome host resistance, it is essential to explore, identify, and utilize novel resistance mechanisms for breeding resilient genotypes [11]. Controlling PMMoV is essential to promote the worldwide development of pepper production. To help control the negative impacts of PMMoV on pepper production, this review presents an overview of the nature of the pathogen in terms of its distribution, economic impacts, management, and genome sequence variation of different isolates, as well as available genetic resources for future breeding.

## 2. The Genus *Tobamovirus*

**Figure 1:**
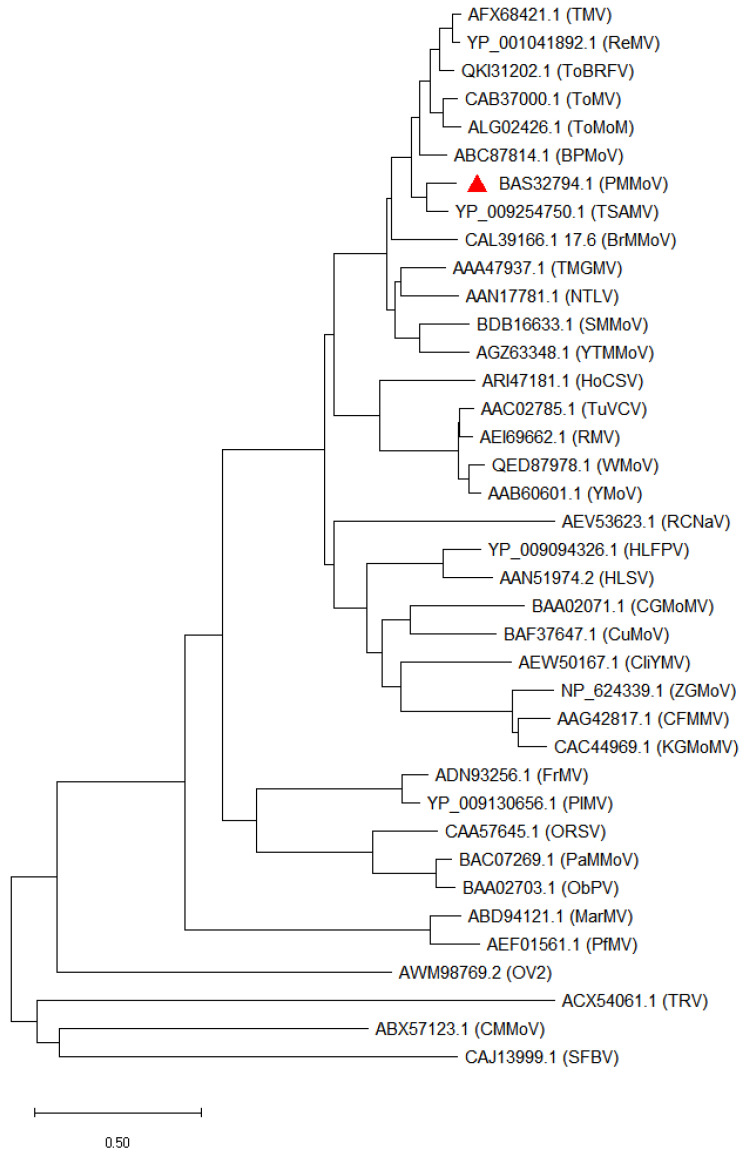
Unrooted phylogenetic tree of a representative isolate of pepper mild mottle virus (PMMoV) and other tobamoviruses based on sequences of their coat proteins (CPs). All the sequences were downloaded from National Center for Biotechnology Information (NCBI) genome database (https://www.nvbi.nlm.nih.gov/) using the corresponding accession numbers. The phylogenetic treewas constructed using MEGAX software. The red colored triangleindicatesPMMoV isolate. The representative isolates are indicated in parentheses against the corresponding protein accession numbers.

Among the various genera, the tobamoviruses include several well-characterized plant viruses (Table 1) containing a positive-sense single-stranded RNA (ssRNA+) genome [9]. The genome of tobamoviruses has a 5′-capped RNA containing four open reading frames that encode a replication protein (~130 kDa), a read-through product (~180 kDa), nonstructural cell-to-cell movement proteins (30 kDa), and a coat protein (CP; 17.5 kDa) [10,12]. The encoded proteins vary across different strains andisolates (Table 1). The replication protein has a methyltransferase-like domain responsible for the 5′ capping of progeny RNAs and an RNA helicase-like domain, whereas the read-through protein contains an RNA-dependent RNA polymerase-like (RdRp) domain [13,14]. The genus *Tobamovirus* is evolutionarily diverse and comprises several highly damaging plant viruses, such as PMMoV, cucumber green mottle mosaic virus, and tomato brown rugose fruit virus (Table 1, Figure 1).

## 3. Genome Sequence Variationof PMMoVIsolates

**Figure 2:**
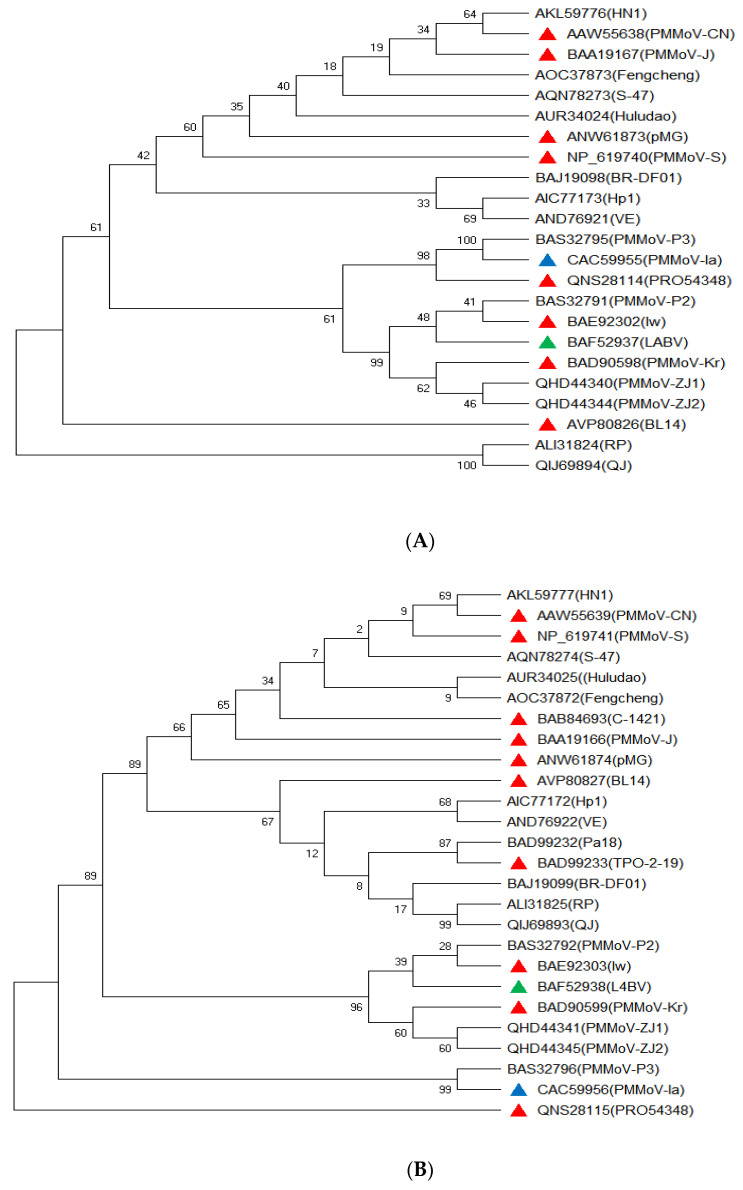

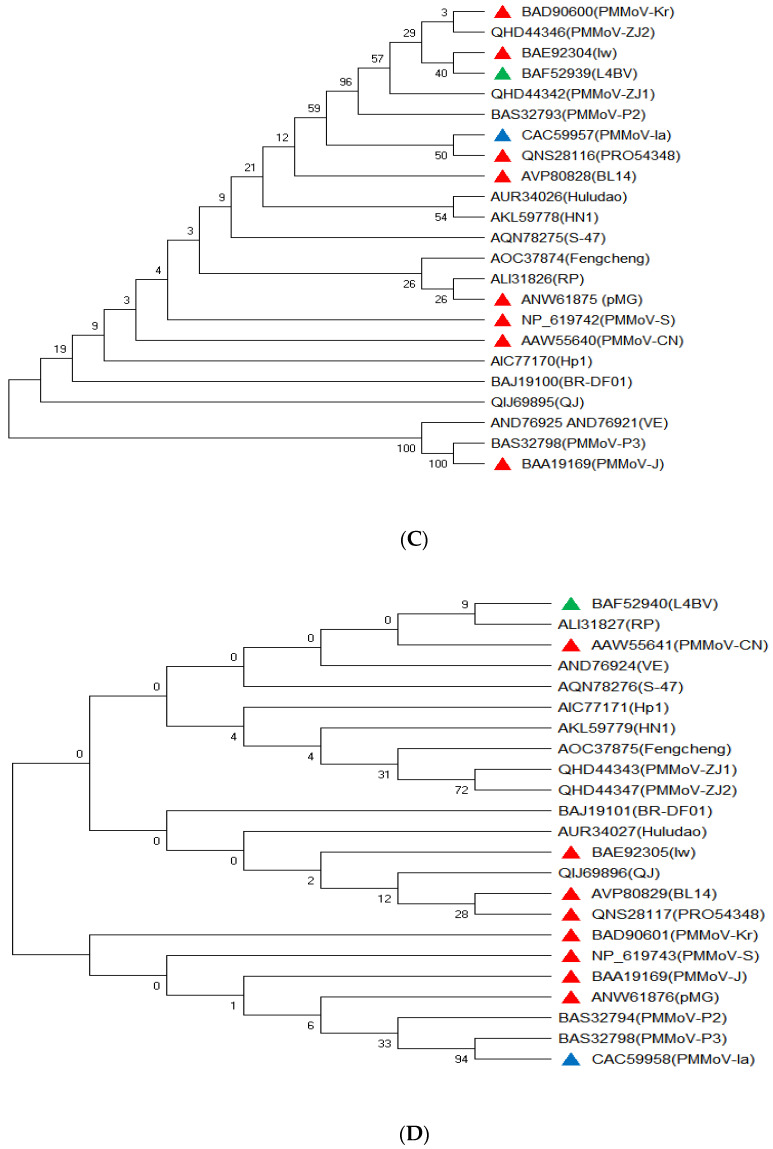
Phylogenetic tree showing the relationships among isolates of PMMoV based on sequences of RNA-dependent RNA polymerase (RdRp) ((**A**), 183 kDa), read-through protein ((**B**), 126 kDa), movement protein ((**C**), 30 kDa), and coat proteins ((**D**), 17 kDa). The protein sequences were downloaded from the NCBI genome database (https://www.nvbi.nlm.nih.gov/) and used forphylogenetic tree conduction using the MEGAXsoftware. The red, blue, and green triangles indicate examples of P1.2; P1.2.3, and P1.2.3.4 pathotypes, respectively.

PMMoV has a monopartite genome [51], consisting of a single RNA molecule that is protected in a shell or capsid. The capsid is composed of proteins that form a the rod-shaped virion of the virus [7]. The virion contains a nonenvelopedssRNA+ genome [52,53]. The first complete genome sequence of PMMoV (Isolate: PMMoV-S) was reported in 1991 [54]. Several experiments have since been conducted to provide information about the complete genome sequencing of PMMoV isolates (Table 2, Figure 2). Complete genome sequencing projects of PMMoV have revealed the presence of variation among different isolates in terms of nucleotide sequence length (Table 2). The genome of PMMoV encodes four different types of proteins: replication-associated proteins (126 kDa and 183 kDa), a movement protein (30 kDa), and a CP (17 kDa) [10,52,55]. Given the challenges regarding the mechanisms of controllingplant viruses, their dynamic and evolvability nature, it is vital to understand the evolutionary relationships among different isolates of the PMMoV pathogen. This knowledge will provide important information for understanding the genetic or mutations associated with resistance-breaking ability of PMMoV strains or isolates, and efficiency of diagnostic tools to use. In addition, knowledge on evolutionary relationships among different pathogen isolates is significant for disease management, germplasm evaluation and crop breeding. Phylogenetic trees based on sequences of coat proteins of different PMMoV isolates whose genomes have been completely sequenced are shown in Figure 2.

## 4. Host Range and Symptoms Associated with PMMoV

**Figure 3:**
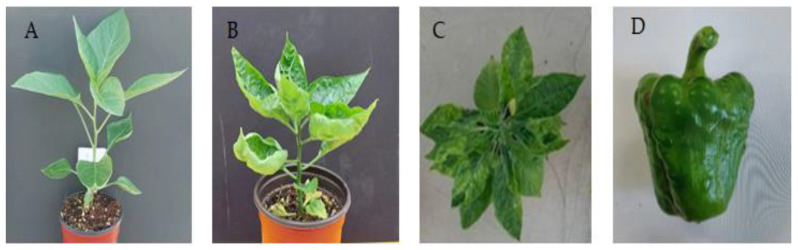
Features of healthy and PMMoV-infected pepper plants. (**A**) Uninfected plant. (**B**) PMMoV-infected plant with mottled leaves. (**C**) PMMoV-infected plant with chlorotic leaves. (**D**) Fruit showing typical symptoms of PMMoVinfection (e.g., blistered and lumpy).

Members of the genus *Tobamovirus* are capable of infecting different Solanaceae species, including tomato, tobacco, and eggplant. Although PMMoV infects species of pepper and tobacco, other susceptible hosts, such as *Dracaenabraunii* [73], tomato [1], and *Parispolyphylla* var. yunnanensis [72], have been reported. Symptomsof infection by PMMoV occurmainly on leaves and fruits (53). At the early stage of infection, plants generally show mild foliar mosaicism. Infected plant leaves subsequently develop mottled, deformed, and chlorotic features [74] (Figure 3A–C). In severe infections, especially those occurring at the early stage of growth, affected plants become stunted, resulting in reduced yields [55]. Fruits of affected plants are decreased in size and appear deformed, necrotic, mosaic, and lumpy or blistered (Figure 3D), thus reducing their market value. The symptoms of PMMoV-affected plants are usually more prominent when infection occurs at the early stages of plant growth. Severe yield losses are likely to occur if an infection is not detected early [59].

## 5. Mode of PMMoV Transmission in Plants

PMMoV can be transmitted mechanically via contact with contaminated sources, including working equipment such as gloves and workers’ clothing, during normal crop management. The pathogen is both seed- and soil-borne and can be transmitted through the use of contaminated seeds in planting as well as planting in infected soils. On seeds, PMMoV occurs on the outer coat of the seed and is transmitted nonembryonically [69]. As the pathogen is seed-borne, it can be easily introduced across different environments, which is likely the reason for the spread of the pathogen in many pepper-growing areas. Wounds and microscopic abrasions on seeds or plant parts facilitate viral entry into the host. PMMoV can remain stable and serve as inocula when adsorbed on plant debris (leaves, stems, and roots), soil, humus, greenhouse structures, and working tools for prolonged periods.

## 6. Global Distribution and Economic Importance of PMMoV

Overall, plant viral diseases account for considerable yield losses and the severity of their impacts on crop production can be accelerated by changing patterns of climate, international trade, and pathogen adaptation for rapid evolution [75]. Globally, annual crop yield losses resulting from plant viruses in the form of small-scale yield reductions to total crop failure havebeen estimated to have a value of more than USD 30 billion [76]. In the early half of the 1950s, PMMoV was described for the first time as a latent strain of tobacco mosaic virus in the USA [77]. In 1984, PMMoV was first described in the literature by an Italian group as a distinct virus [78]. Currently, disease incidences associated with the pathogen are spread across different parts of the world (Table 2). PMMoV is particularly adapted to survive under extreme conditions, such as warm, hot, and humid climates. The fast-spreading nature of the pathogen poses a serious threat to pepper cultivation and food security. In addition, the broad scope of PMMoV isolates is indicative of the pathogen’s exceptional adaptation, which may enable some strains to easily overcome known resistance genes and even expand their host range. Meanwhile, the severity of impacts resulting from PMMoV infection in pepper differs according to the isolate, host species, and the stage of plant growth during which the infection occurs. The disease incidence resulting from PMMoV infection in commercial bell pepper fields in Florida, USA varied from <1% to 30% [79]. In another study in Grady County, Georgia, USA, an entire jalapeno pepper (*Capsicum annuum* L.) field was reported to have been devastated by PMMoV [80]. Fruits of infected plants were deformed, mottled, reduced in size, and had off-colored sunken parts.

## 7. Management of Pepper Mild Mottle Virus (PMMoV)

Diseases originating from PMMoV infections are exceptionally difficult to control when they occur. Once infected with PMMoV, it becomes extremely difficult to recover plants using chemical or physical treatments [81]. Therefore, to control infections with and the spread of PMMoV, growers must observe good cultural and sanitation practices in their production systems. Avoiding sources of infection by the disinfection of working tools and removal and destruction of infected plants can help to control the spread of the pathogen. Working tools or equipment and stakes must be disinfected to minimize possible transmission. The pathogen is seed-borne; therefore, clean seeds must be used to establish pepper production. Sanitary certification and cross-protection management are important factors to control PMMoV. Care must also be taken to avoid abrasions and induced wounds on plant parts, as this is ideal for viral entry into host tissues. It is also essential to gain an understanding of which weed species can act as hosts of the virus. In addition, the genetic relationships among existing strains of PMMoV must be studied to facilitate accurate diagnosis in research. Knowledge about causal agents and symptoms associated with the virus as well as regular inspection of plants for the early detection of PMMoV infection are required. Under less severe conditions, plants showing noticeable symptomsof viral infection and adjacent plants should be removed immediately, but care must be taken to avoid touching healthy plants with contaminated hands or tools. Rotation with resistant crops and sterilizing soils before planting for greenhouse cultivation are good practices to break the disease cycle via inocula from plant debris [82]. The planting of resistant pepper genotypes is also highly recommended.

## 8. Genetic and Gene Resources for Resistance Breeding against PMMoV

### 8.1. Diversity of L Resistance Genes and PMMoV Pathotypes

Due to the rapid spread and damaging nature of PMMoV, it is necessary to incorporate resistance alleles from PMMoV-resistant cultivars into the genome of commonly cultivated peppers. The *L* genes (*L1*–*L4*) that functionally control resistance of peppers to tobamovirusesdiffers across different pepper species and cultivars. Based on differences in the L gene allele, *Capsicum* species have been divided into *L1*, *L2*, *L3*, and *L4* classes [10]. On the other hand, tobamoviruses are classified into four pathotypes—P0, P1, P1.2, and P1.2.3—according to their ability to systemically infect *Capsicum* species carrying *L* gene alleles *L1*, *L2*, *L3*, and *L4*, respectively [10,83]. Following infection, the PMMoV CP induces expression of the *L* genes and thus induces a HR in the host plant [84]. Two *L* gene alleles, *L3* and *L4*, confer high degrees of resistance to PMMoV. Pepper plants harboring the *L3* gene show resistance to the P1.2 pathotype but are susceptible to P1.2.3. Plants that have the *L4* geneshow resistance to these two pathotypes and have a broader scope of resistance against tobamoviruses. The crop germplasm represents a significant tool for identifying disease resistance genotypes for crop breeding. Extensive screening of diverse accessions to identify PMMoV-resistant resources has been conducted using various gene markers (Table 3). Although commercial pepper varieties have been developed that carry *L* genes that confer resistance to the pathogen, there are strains of PMMoV that can overcome some of these resistance genes. Moreover, previous studies revealed that many host plant species harbor specific genes encoding a protein known as the TOBAMOVIRUS MULTIPLICATION (TOM) susceptibility protein, which interacts with the viral replication protein [85]. The mechanism underlying the host plant–viral protein interaction favorably promotes certain *Tobamovirus* replication complexes and pathogen multiplication, and leads to effective infectivity on the host. Molecular studies usingthe CRISPR/Cas9-derived suppression of certain host TOM-related genes have been successfully conducted in different plant species, including *Arabidopsis*, tomato, and tobacco [85], but this technique has not been fully explored for resistance breeding against PMMoV.

### 8.2. Pepper Genetic Resources with Resistance against PMMoV

**Figure 4:**
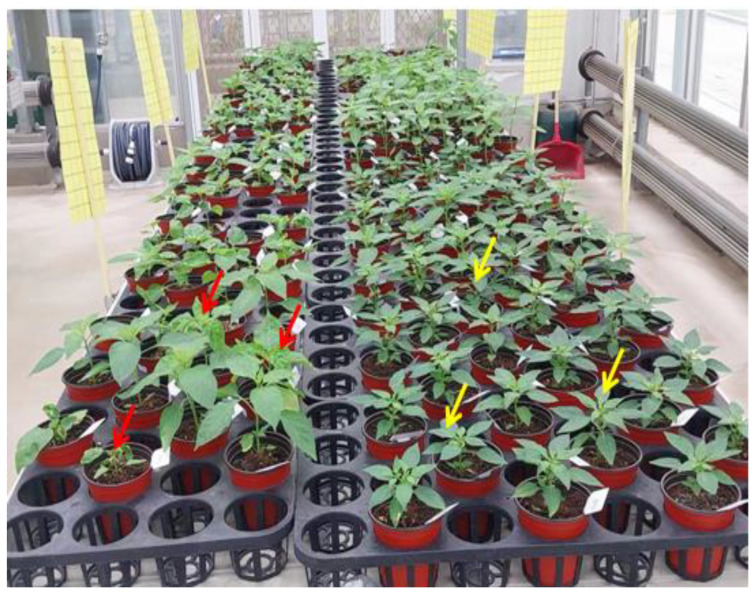
Mechanical inoculation of pepper germplasm with PMMoV pathogen under greenhouse conditions. Red and yellow arrows indicate plants of susceptible and resistant accessions, respectively. The photographs were taken 14 days postinoculation (DPI) during greenhouse experimental screening.

Pepper cultivation is widely distributed across the globe, with specific selections conducted over the past several years in different locations, resulting in differences in cultivar adaptation potential in diverse environments. Although pepper has a wide range of cultivars, many cultivated varieties display susceptibility to PMMoV [90]. Germplasm screening is an important means of identifying *Capsicum* accessions that may contain genes capable of conferring resistance to the virus. Nonetheless, the resistance of *Capsicum* species to PMMoV differs considerably across different genotypes (Figure 4), with some genotypes succumbing easily to specific strains but showing resistance to other strains (Table 4). This phenomenon can be attributed to the presence of variability in strains or isolates of the pathogen. A comprehensive characterization that combinesphenotyping and genotyping strategies isrequired to identify useful PMMoV-resistant accessions [81]. Thus, pepper breeders screened diverse pepper germplasm resources and identified some accessions with resistance against different PMMoVpathotypes that are useful for further breeding (Table 4).

## 9. PMMoV as an Indicator of Water Quality

Pepper is the most important spicy vegetable crop, with worldwide consumption of its fresh fruits and processed dry powder, as wellas an ingredient in some industrial consumable products. Strategies to control PMMoV in pepper fields will help to increase fruit yield and reduce the likelihood of water contamination [7]. There are research studies that have confirmed the detection of PMMoV in domestic and aquatic water resources, as well as in the excreta of animals, including humans (Table 5). The pathogen is known to have survival ability in the human gastrointestinal tract, and thus can be transmitted via human excreta [92]. Human immune response and clinical symptoms linked to PMMoV infection have also been investigated experimentally and include fever, abdominal pains, and pruritus (7). Though this finding may be possible, the detected symptoms may also result from other cofactors [93]. The key source of the pathogen’s presence in human excreta has been attributed largely to the consumption of PMMoV-infected pepper and its processed products, while human excreta-derived pollution of water resources is the reason for the presence of the virus in water bodies [94,95,96]). Therefore, the presence of PMMoV in water is currently considered an indicator for water quality assessment [8,97,98,99]. Moreover, the stable nature of PMMoV in water raises additional concerns in relation to their possible transmission via contaminated irrigation water resources [92,100,101].

## 10. Conclusions

PMMoV is an economically significant pathogen responsible for yield losses in pepper production, and poses a serious threat to agriculture and food sustainability. Detection of the virus in water resources, including aquatic, irrigation, pond, underground, and domestic water, as well as in the excreta of animals, including humans, raises additional concerns requiring extensive research to develop solutions that can circumvent potential risks associated with the pathogen in relation to human health. The application of molecular breeding techniques has prospects for the development of new cultivars that are resilient against PMMoV. Mutagenesis, including physical, chemical, and biological techniques, can be used in combination with next-generation sequencing to explore and exploit beneficial candidate genes for molecular breeding targeting the development of PMMoV-resistant genotypes.

## Figures and Tables

**Table 1:** Plant viruses belonging to the genus *Tobamovirus* of the family *Virgaviridae*.

Name of Virus	Short Name	Representative Isolate	GeneBank Accession	Nucleotide Length (kb)	Host Common Name	Host Taxonomic Name	Host Family	Reference
Pepper mild mottle virus	PMMoV	PMMoV-p2	LC082099	6.356	Paprika	*Capsicum annuum*	Solanaceae	[10]
Bell pepper motile virus	BPMoV	BPMoV-P1	DQ355023	6.375	Eggplant	*Solanum melongena*	Solanaceae	[15]
Paprika mild mottle virus	PaMMoV	PaMMoV-J	ABO89381	6.525	Sweet pepper	*Capsicum annuum* L.	Solanaceae	[16]
Obuda pepper virus	ObPV	ObPV-Ob	D13438	6.507	Tobacco	*Nicotiana tabacum* cv. Xanthi nc.	Solanaceae	[17]
Tomato mosaic virus	ToMV	S1	AJ132845	6.384	Tomato	*Solanum* spp.	Solanaceae	[18]
Tomato brown rugose fruit virus	ToBRFV	ToBRFV-CA18-01	MT002973	6.389	Tomato	*Solanum lycopersicum*	Solanaceae	[19]
Tomato mottle mosaic virus	ToMoMV	ToMMV_NY-13	KT810183	6.398	Tomato	*Solanum lycopersicum*	Solanaceae	[20]
Tobacco mosaic virus	TMV	TMV-variant 1	V01408	6.395	Tobacco	*Nicotiana* spp.	Solanaceae	[21]
Tobacco mild green mosaic virus	TMGMV	-	M34077	6.355	Tobacco	*Nicotiana tabacum*	Solanaceae	[22]
Tobacco rattle virus	TRV	TRV MI-1 RNA-1	GQ903771	6.791	Potatoes		Solanaceae	[23]
Tobacco latent virus	NTLV		AY137775	1.415	Tobacco	*Nicotiana* species	Solanaceae	[24]
Brugmansia mild mottle virus	BrMMoV	BrMMoV-2373	AM398436	6.381	Brugmansia	*Angel’s trampet*	Solanaceae	[25]
Tropical soda apple mosaic virus	TSAMV	TSAMV-Okeechobee	NC030229	6.350	Tropical soda apple	*Solanum viarum*	Solanaceae	[26]
Scopolia mild mottle virus	SMMoV	SMMoV-Kyo35	LC643028	6.350	Japanese belladonna	*Scopolia japonica*	Solanaceae	[27]
Yello tailflower mild mottle virus	YTMMoV	YTMMV-Cervantes	KF495564	6.379	Yellow tailflower	*Anthocercislittorea*	Solanaceae	[28]
Cucumber fruit mottle mosaic virus	CFMMV	CFMMV	AF321057	6.562	Cucumber	*Cucumis sativus*	Cucubitaceae	[29]
Cucumber green mottle mosaic virus	CGMoMV	CGMMV-SH	D12505	6.424	Muskmelon	*Cucumis melo*	Cucubitaceae	[30]
Cucumber mottle virus	CuMoV	CMoV	AB261167	6.485	Cucumber	*Cucumis sativus*	Cucubitaceae	[31]
Kyuri green mottle mosaic virus	KGMoMV	KGMoMV-C1	AJ295948	6.514	Cucumber	*Cucumis sativus* L.	Cucubitaceae	[32]
Zucchini green mottle mosaic virus	ZGMoV	ZGMMV-K	NC003878	6.513	Zucchini squash	*Cucurbita pepo* L. zucchini	Cucubitaceae	[33]
Turnip vein-clearing virus	TuVCV	TuVCV-OSU	U03387	6.311	Turnip	*Brassica rapa*	Brassicaceae	[34]
Wasabi mottle virus	WMoV	WMoV-SFU2	MK431779	6.297	Wasabi	*Wasabi japonica* (Miq) Matsum	Brassicaceae	[35]
Youcai mosaic virus/Oilseed rape mosaic virus	YMoV		U30944	6.303	Rapeseed	*Brassica napus*	Brassicaceae	[36]
Hibiscus latent Fort Pierce virus	HLFPV	HLFPV-J	NC025381	6.431	Hibiscus	*Hibiscus* spp.	Malvaceae	[37]
Hibiscus latent Singapore virus	HLSV	Singapore	AF395898	6.485	Hibiscus	*Hibiscus rsa-sinensis*	Malvaceae	[38]
Clitoria yellow mottle virus	CliYMV	Larrimah	JN566124	6.514	Butterfly peas	*Clitoriaternatea*	Fabaceae	[39]
Sunn-hemp mosaic virus	SHMV	SHMV	U47034	4.683	Sunn-hemp	*Crotalaria juncea*	Fabaceae	[40]
Cactus mild mottle virus	CMMoV	CMMoV-Kr	EU043335	6.449	Diseased grafted cactus	*Gymnocalyciummihanovichii*	Cactaceae	[41]
Opuntia virus 2	OV2	Nopal_hec Mex	MF434821	6.4.53	Prickly pear (mixed sample)	*Opuntia* sp.	Cactaceae	[12]
Rattail cactus necrosis-associated virus	RCNaV	RCNaV	JF729471	6.506	Ratilcatus	*Aporocactusflagelliformis*	Cactaceae	[42]
Frangipani mosaic virus	FrMV	FrMV-P	HM026454	6.643	Frangipani	*Plumeria rubra* f. acustifolia	Apocynaceae	[43]
Maracuja mosaic virus	MarMV	MarMV	DQ356949	6.794	Passion fruit	*Passiflora edulis* Sims ‘Flavicarpa’	Passifloraceae	[13]
Passion fruit mosaic virus	PfMV	PfMV	HQ389540	6.791	Passion fruit	*Passiflora incarnata* L.	Passifloraceae	[44]
Odontoglossum ringspot virus	ORSV	ORSV-Cy	X82130	6.618	Tobacco	*Nicotiana tabacum*	Orchidaceae	[45]
Plumeira mosaic virus	PlMV	Plu-Ind-1	NC026816	6.688	Frangipani	*Plumeria rubra f. acustifolia*	Apocynaceae	[46]
Rehmannia mosaic virus	ReMV	Henan	NC009041	6.395	Rehmannia	*Rehmanniaglutinosa* Libosch	Orobanchacee	[47]
Ribgrass mosaic virus	RMV	Kons 1105-R14	HQ667979	6.311	Rigbgrass	*Plantago major* L.	Plantaginaceae	[48]
Streptocarpus flower break virus	SFBV	SFBV	AM040955	6.279	Streptocarpus	*Streptocarpus* spp.	Gesneriaceae	[49]
Hoya chlorotic spot virus	HoCSV	12-415	KX434725	6.386	Hoya wayetii	*Hoya* spp.	Asclepiadaceae	[50]

**Table 2:** PMMoV isolates with available complete genome sequence information.

PMMoV Isolate	Country	Accession Number	Sequence Length (kb)	Protein ID (183 kDa)	Host	Reference
PMMoV-P2	Republic of Korea	LC082099	6.356	BAS32791	*Capsicum* spp.	[10]
PMMoV-P3	Republic of Korea	LC082100	6.356	BAS32795	*Capsicum* spp.	[10]
S-47	Republic of Korea	KX399390	6.356	AQN78273	*Capsicum* spp.	[55]
J-47	Republic of Korea	KX399389	6.356	AQN78269	*Capsicum* spp.	[55]
PMMoV-Kr	Republic of Korea	AB126003	6.356	BAD90598	*Capsicum* spp.	[56]
PMMoV-Ia	Spain	AJ308228	6.357	CAC59955	*Capsicum* spp.	[57]
BR-DF01	Brazil	AB550911	6.356	BAJ19098	_	[58]
Hp1	India	KJ631123	6.356	AIC77173	*Capsicum* spp.	[59]
VE	Venezuela	KU312319	6.356	AND76921	*Capsicum* spp.	[60]
PMMoV-S	Spain	NC003630	6.357	NP_619740	*Capsicum* spp.	[54]
Huludao	China	MG515725	6.356	AUR34024	*Capsicum* spp.	[53]
HN1	China	KP345899	6.356	AKL59776	*Capsicum* spp.	[61]
PMMoV-CN	China	AY859497	6.356	AAW55638	*Capsicum* spp.	[62]
Fengcheng	China	KU646837	6.356	AOC37873	*Capsicum* spp.	[63]
PMMoV-ZJ1	China	MN616926	6.356	QHD44340	*Capsicum* spp.	[64]
PmmoV-ZJ2	China	MN616927	6.357	QHD44344	*Capsicum* spp.	[64]
PMMoV-J	Japan	AB000709	6.357	BAA19167	*Capsicum* spp.	[65]
Iw	Japan	AB254821	6.356	BAE92302	*Capsicum* spp.	[66]
BL14	USA	MH063882	6.353	AVP80826	*Capsicum* spp.	[4]
C-1421	Japan	AB069853	6.357	BAB84693	*Capsicum* spp.	[67]
Pa18	Japan	AB113116	6.356	BAD99232	*Capsicum* spp.	[68]
TPO-2-19	Japan	AB113117	6.356	BAD99233	*Capsicum* spp.	[68]
PRO54348	Chile	MT385868	6.356	QNS28114	*Capsicum* spp.	[52]
L4BV	Japan	AB276030	6.356	BAF52937	*Capsicum* spp.	[69]
RP	Republic of Korea	KR108206	6.356	AL131824	*Rorippa palustris*	[70]
pMG	Spain	KX063611	6.361	ANW61873	*Capsicum* spp.	[71]
QJ	China	MK784568	6.357	QIJ69894	*P. polyphylla*	[72]

**Table 3:** DNA markers used in the genotype selection of PMMoV-resistant pepper accessions.

Marker	Primer	Primer Sequence (5′–3′)	Primer Size (bp)	Type	Resistance	Reference
	AP-7	CGTACTGTGGCTCAAAACTC	-	-	*L4*	
SCAR	AP-8	ATTCGCACCGTTTAGCCCGT	-	-	*L4*	[86]
	087H3T7150F	CATGATTACATTTTATGTTGC		Co-dominant	*L4*	
087H3T7	087H3T7150R	AAAAGGAAGGTTCTCATTGTT	150		*L4*	[87]
	087H3T7F	CCTTTGCCTGCATTATTCTTG			*L4*	
087H3T7	087H3T7R	GCCCAAATTTATTCCCAAATGC	440	Co-dominant	*L4*	[87]
	060I2END-2F	GCACATCAGCAGGTTTAGTACG			*L4*	
060I2END	060I2END-2R	CCAACTGTCAAACCTCGGTT	751	Co-dominant	*L4*	[87]
	158K24HRMF	CAGATTAAGTGTTCAAAATGAGTGATG		Co-dominant	*L4*	
158K24HRM	158K24HRMR	TGATTCCATGAAAATAAATTGTAAAGA	125		*L4*	[87]
	F	AAGGGGCGTTCTTGAGCCAA		-	*L4*	
L4SC340	R	TCCATGGAGTTGTTCTGCAT	340	-	*L4*	[88]
	PMF1	CTGCAGAACAACAATGGCACG		Co-dominant	*L3*	
PMFR11269	PMR1	GCTTCCTCCTCTGCAGTCC	268		*L3*	[89]
	PMF2	GCCAAAATGGTAATTG		Co-dominant	*L3*	
PMFR11283	PMF1	GCTTCCTCCTCTGCAGTCC	283		*L3*	[89]

**Table 4:** Pepper genetic resources with resistance against PMMoVpathotypes.

Germplasm (Name or Accession) *	Pepper Type	Resistance Genotype	PMMoV Pathotype	Reaction	Response	Screening Method	Reference
Easy	*C. annuum*	L4L4	P1.2 and P1.2.3	NS/-	R	Bioassay and genetic markers	[11]
Magnipico	*C. annuum*	L4L4	P1.2 and P1.2.3	NS/	R	Bioassay and genetic markers	[11]
Orange glory	*C. annuum*	L4L3	P1.2 and P1.2.3	NS/	R	Bioassay and genetic markers	[11]
Scirocco F1	*C. annuum*	L4L3	P1.2 and P1.2.3	NS/	R	Bioassay and genetic markers	[11]
Special F1	*C. annuum*	L4L1	P1.2 and P1.2.3	NS/	R	Bioassay and genetic markers	[11]
IT261210 *	*C. chinense*		PMMoV-1.2.3	Nl/-	R	Bioassay and RT-PCR	[81]
IT261211 *	*C. chinense*		PMMoV-1.2.3	Nl/-	R	Bioassay and RT	[81]
IT261426 *	*C. chinense*		PMMoV-1.2.3	Nl/-	R	Bioassay and RT	[81]
IT261431 *	*C. chinense*		PMMoV-1.2.3	Nl/-	R	Bioassay and RT	[81]
IT261442 *	*C. chinense*		PMMoV-1.2.3	Nl/-	R	Bioassay and RT	[81]
IT284050 *	*C. chinense*		PMMoV-1.2.3	Nl/-	R	Bioassay and RT	[81]
PI 152225 *	*C. chinense*	L3	PMMoV-P1.2	Nl/	R	Mechanical and biological characterization	[91]
PI 260429 *	*C. chinense*	L4	PMMoV-P1.2	Nl/	R	Mechanical and biological characterization	[91]
PI260429 *	*C. Chacoense*	L4	PMMoV-1.2.3	-	R	SCAR DNA marker	[86]
SA185 *	*C. Chacoense*	L4	PMMoV	-	R	SCAR DNA marker	[86]
Susan	*C. annuum*	L4	PMMoV	-	R	SCAR DNA marker	[86]
Special	*C. annuum*	L4	PMMoV-1.2.3	-	R	SCAR DNA marker	[86]
AP-PM01 *	*C. annuum*	L4	PMMoV	-	R	SCAR DNA marker	[86]
AP-PM02 *	*C. annuum*	L4	PMMoV	-	R	SCAR DNA marker	[86]
AP-PM03 *	*C. annuum*	L4	PMMoV-1.2.3	-	R	SCAR DNA marker	[86]
AP-PM04 *	*C. annuum*	L4	PMMoV	-	R	SCAR DNA marker	[86]
AP-PM05 *	*C. annuum*	L4	PMMoV	-	R	SCAR DNA marker	[86]
AP-PM06 *	*C. annuum*	L4	PMMoV	-	R	SCAR DNA marker	[86]
Kyouyutaka	*C. annuum*	L1	PMMoV-1.2	-	R	SCAR marker	[89]
Tosahikari D		L1	PMMoV-1.2	-	R	SCAR marker	[89]
Tabasco	*C. frutescens*	L2	PMMoV-1.2/1.2.3	-	R	SCAR marker	[89]
PI159236 *	*C. chinense*	L3	PMMoV-1.2/1.2.3	-	R	SCAR marker	[89]
Berumasari	*C. annuum*	L3	PMMoV-1.2/1.2.3	-	R	SCAR marker	[89]
Himukamidori	*C. annuum*	L3	PMMoV-1.2/1.2.3	-	R	SCAR marker	[89]
T-143 *	*C. annuum*	L3	PMMoV-1.2/1.2.3	-	R	SCAR marker	[89]
Tosahime R	*C. annuum*	L3	PMMoV-1.2/1.2.3	-	R	SCAR marker	[89]
Spirit	*C. annuum*	L3	PMMoV-1.2/1.2.3	-	R	SCAR marker	[89]
Mihata 1	*C. annuum*	L3	PMMoV-1.2/1.2.3	-	R	SCAR marker	[89]
Sarara	*C. annuum*	L3	PMMoV-1.2/1.2.3	-	R	SCAR marker	[89]
Miogi	*C. annuum*	L3	PMMoV-1.2/1.2.3	-	R	SCAR marker	[89]
Fiesta	*C. annuum*	L3	PMMoV-1.2/1.2.3	-	R	SCAR marker	[89]
PI260429	*C. chacoense*	L4	PMMoV-1.2/1.2.3	-	R	SCAR marker	[89]
Leira	*C. annuum*	L4	PMMoV-1.2/1.2.3	-	R	SCAR marker	[89]
Special	*C. annuum*	L4	PMMoV-1.2/1.2.3	-	R	SCAR marker	[89]

* Refers to pepper accessions.

**Table 5:** Research findings on the detection of PMMoV in water resources and in animal (including human) excreta.

Study Location	Year	Sample Source	Detection Method	Key Findings	References
Atlantic, USA	2021	Surface and reclaimed water	RT-qPCR	1. PMMoV detected more in reclaimed water than in surface water samples. 2. Water salinity affected the detection of PMMoV and other enteric viruses.	[102]
Japan	2021	Surface and tap water	RT-qPCR and SD-CDDP-(RT-qPCR	1. PMMoV detected in surface water. 2. Intact PMMoV was more common than intact human pathogenic viruses.	[103]
Italy	2021	Raw and treated sewage, river, estuarine, bathing water, groundwater, and drinking water	Nested RT-qPCR and sequencing	1. PMMoV detected in both treated and untreated sewage, river, estuarine water, bathing water, and groundwater samples. 2. No PMMoV detected in drinking water.	[104]
Costa Rica	2021	River and ocean discharge sites	RT-qPCR	1. PMMoV and HF183 detected in all river samples and in >89% of ocean samples.	[105]
Slovenia	2021	PMMoV -containing plant homogenates and PMMoV-free homogenates	Test plant infectivity assays, transmission electron microscopy, RT-PCR- and RT-droplet digital PCR	1. PMMoV is a very resilient water-transmissible *Tobamovirus* and can survive transit through the human gut. 2. CAP is a useful water treatment tool for inactivation of pathogenic viruses, including PMMoVand other enteric viruses.	[92]
Japan	2021	Groundwater (well water)	Quantitative microbial risk assessment, membrane filtration method	1. PMMoV detected in well water in high based only on the horizontal distance as the PMMoV concentration decreased rapidly as distance increased.	[100]
Italy	2020	Urban wastewaters, treated effluents, surface water, estuarine, seawater, groundwater, and drinking water	Nested RT-PCR and TaqMan-based qPCR	1. PMMoV detected in wastewater, treated sewage, river, estuarine, bathing water, and groundwater samples. 2. No PMMoV detected in drinking water samples. 3. PMMoV is ubiquitous throughout the water cycle with different concentrations.	[106]
Mexico	2020	Fecal, oropharyngeal (gastrointestinal) samples.	NextSeq500 Illumina platform	1. PMMoV, in addition to tropical soda apple mosaic virus and opuntia virus 2, were the most common species detected in fecal and oropharynx samples.	[107]
Slovenia	2020	Influents and effluents ofwastewater treatment plants	RT-qPCR	1. High-diversity plant viruses, especially tobamoviruses, were detected in wastewater treatment plant influents and effluents.	[101]
Japan	2020	Human enteric viruses and PMMoV	PMA-PCR, PMA-Enhancer-PCR, PMAxx-PCR, and PMAxx-Enhancer-PCR	1. PMMoV was more resistant to heat treatments and could be a potential surrogate for some enteric viruses in thermal disinfection processes. 2. The PMMoV was comparatively much more resistant to chlorine treatment.	[108]
Egypt	2020	Influent and effluentwastewater	g qRT-PCR	1. PMMoV was detected in both influent and effluent samples and no clear seasonality of detection was found. 2. PMMoV can be used as a fecal indicator of wastewater contamination and a process indicator for the performance of the treatment process.	[109]
Nepal	2019	Tanker water	qPCR	1. PMMoV together with tobacco mosaic virus was detected in tanker water.	[110]
Kenya	2019	Wastewater and wastewater-impacted surface waters	RT-PCR	1. PMMoV and other enteroviruses were detected in all samples and could be used as indicators in fecal contaminated sites.	[111]
New Zealand	2019	Nonhuman fecal matter; influent wastewater, and fish-growing waters	RT-qPCR	1. Certain nonhuman fecal samples (seagull, Canada goose, black swan, and dog) were positive for PMMoV. 2. PMMoV detected in shellfish and shellfish-growing water samples.	[112]
Japan	2018	Surface water	Conventional plaque assay, RT-qPCR	1. PMMoVdetected in the surface water samples regardless of season and location, and is useful as an indicator for water contamination.	[113]
Japan	2018	Water	Taqman-based RT-qPCR	1. PMMoV detected in raw water throughput the year and can serve as a treatment process indicator of enteric viruses.	[114]
Singapore	2018	Water from different water bodies	Hollow fiber ultrafiltration, ImProm-II reverse transcription system (Promega), qPCR	1. PMMoV detected in the water sample and can be used as a suitable indicator of fecal pollution in tropical surface waters.	[115]
Kathmandu Valley, Nepal.	2018	Irrigation water sources	Electronegative membrane-vortex method and TaqMan (MGB)-based qPCR assays	1. PMMoV (and TMV) detected in all types of irrigation water sources and is a potential indicator to elucidate pathogenic virus levels in environmental samples. 2. Seasons had good correspondence with the presence of pathogenic virus types.	[95]
Costa Rica	2017	Fecal matter of animals, domestic wastewater, and surface water	RT-qPCR	1. PMMoV is a useful domestic wastewater-associated marker, with high concentrations and 100% sensitivity and specificity. 2. PMMoV markers were not detected in any surface water samples.	[116]
Mexico	2017	Groundwater	RT-PCR and cloning	1. PMMoV RNA detected in most samples with gene sequences sharing 99–100% of nucleotide identity with other PMMoV sequences. 2. No significant correlation observed between PMMoV occurrences by season or water type.	[117]
Southeastern Florida	2016	Surface water samples from inlets, exposed to runoff and septic seepage, and coastal sites, exposed to ocean outfalls	RT-qPCR	1. PMMoV detected more frequently than other microbial source tracking markers.	[118]
Southern Arizona	2016	Wastewater	TaqMan-based qPCR	1. PMMoV in addition to AiV, AdV, JCPyV and BKPyV were detected in the samples and are potential viral markers for human fecal contamination. 2. Frequency of PMMoV detection was less influenced by seasonal variation.	[119]
Hanoi, Vietnam	2015	Surface water, wastewater, groundwater, tap water, and bottled water	qPCR	1. PMMoV detected in many surface water samples and in all wastewater samples in high concentration. 2. PMMoV is useful as a sensitive fecal indicator for evaluating the potential occurrence of pathogenic viruses. 3. No PMMoV detection in tap water and bottled water samples.	[97]
Southern Arizona	2014	Wastewater samples	RT-qPCR TaqMan-based quantitative PCR (qPCR) assays	1. PMMoV (and AiV) detected in both influent and effluent water. 2. PMMoV can be used as potential indicator of wastewater reclamation. 3. No significant seasonal change in concentration of PMMoV was recorded.	[120]
Japan	2013	Drinking water sources	qPCR	1. Significant difference in the occurrence of PMMoV observed among geographical regions but not a seasonal difference. 2. PMMoV strains were diverse in the water sources.	[98]
Germany	2011	Rivers, influents and effluents of wastewater; animal (including human) stool.	Quantitative real time (RT-) PCR	1. PMMoV highly detected in all river water samples, while frequently of other viruses (HAdV and HPyV, TTV and hPBV) were less detected. 2.PMMoV could be a promising indicator of fecal pollution in surface water.	[99]
USA	2010	Commercialized food products containing peppers; human stool	RT-PCR, sequencing, and electron microscopy	1. PMMoV in feces can infect host plants and is viable after passing through the gut. 2. Individuals (humans) positive for PMMoV showed symptoms such as pain in the stomach and mild fever.	[93]
USA	2009	Raw sewage, treated wastewater, seawater exposed to wastewater, and fecal samples and intestinal homogenates from a wide variety of animals	qPCR	1. PMMoV was present in all wastewater and some seawater samples but at higher concentrations in raw sewage and has a potential utility as an indicator of human fecal pollution. 2. Though ubiquitous in human feces, PMMoV was not detected in the majority of animal fecal samples tested (except chicken and seagull samples). 3. PMMoV was not found in nonpolluted seawater samples but could be detected in surface seawater.	[8]
San Diego, California, United States	2006	Fecal samples from two healthy human individuals	RT-PCR	1. PMMoV detected in human fecal samples and high concentration of its viron particles observed in the samples. 2. The vast majority of the viral sequences showed similarity to plant pathogenic RNA viruses. 3. PMMV was also detected in some fecal samples from healthy individuals. 4. A number of pepper-based foods were tested positive for PMMV, which suggests a dietary origin for the virus. 5. PMMV derived from fecal matter is infectious to host plants.	[96]

RT-ddPCR: RT-droplet digital PCR; CAP: cold atmospheric plasma for waterborne virus inactivation; PMAxx: improved PMA; HAdV: human adenovirus, a recognized indicator for human fecal contamination; AiV: Aichi virus; JCPyV/BKPyV: human polymaviruses; hPBV: human picobirnaviruses; TTV: torque teno virus; HpyV: human polyomaviruses; EVs: enteric viruses; AdV: adenovirus type 40; CV: coxsackievirus B5; HF183: microorganism (bacteroides).

## Data Availability

Not applicable.
